# Recurrent Respiratory Papillomatosis: HPV Genotypes and Risk of High-Grade Laryngeal Neoplasia

**DOI:** 10.1371/journal.pone.0099114

**Published:** 2014-06-11

**Authors:** Turid Omland, Kathrine A. Lie, Harriet Akre, Lars Erik Sandlie, Peter Jebsen, Leiv Sandvik, Dag Andre Nymoen, Davit Bzhalava, Joakim Dillner, Kjell Brøndbo

**Affiliations:** 1 Department of Otorhinolaryngology/Head and Neck Surgery, Oslo University Hospital and University of Oslo, Institute of Clinical Medicine, Oslo, Norway; 2 Department of Pathology, Oslo University Hospital, Oslo, Norway; 3 Department of Biostatistics, Oslo University Hospital and University of Oslo, Faculty of Dentistry, Oslo, Norway; 4 Departments of Laboratory Medicine, Medical Epidemiology & Biostatistics, Karolinska Institute and Department of Clinical Microbiology and Pathology, Karolinska Hospital, Stockholm, Sweden; Duke Cancer Institute, United States of America

## Abstract

Patients with recurrent respiratory papillomatosis (RRP) in Norway treated between 1987 and 2009 were recruited to this cohort study. They were followed from disease onset and data recorded until January 2012. Here, we describe the distribution of human papillomavirus (HPV) genotypes, the prevalence of multiple HPV infections, and the risk of high-grade laryngeal neoplasia and respiratory tract invasive carcinoma in a large cohort of patients with RRP. We also examined whether HPV genotype, gender, age or clinical course are risk factors for this development. Clinical records and histological specimens were reviewed. Using formalin-fixed paraffin-embedded biopsies, HPV genotyping were performed by quantitative polymerase chain reaction assays identifying 15 HPV types. HPV-negative specimens were analyzed by metagenomic sequencing. Paraffin blocks were available in 224/238 patients. The DNA quality was approved in 221/224 cases. HPV DNA was detected in 207/221 patients and all were HPV 6 or HPV 11 positive, comprising HPV 6 in 133/207, HPV 11 in 40/207 cases and HPV 6/11 in 15/207 cases. Co-infection with one or two high-risk HPV types together with HPV 6 or HPV 11 was present in 19/207 patients. Metagenomic sequencing of 14 HPV-negative specimens revealed HPV 8 in one case. In total, 39/221 patients developed high-grade laryngeal neoplasia. 8/221 patients developed carcinoma of the respiratory tract (six patients with laryngeal carcinoma and two patients with lung carcinoma). High-grade laryngeal neoplasias were found more frequently in HPV-negative versus HPV-positive patients, (RR = 2.35, 95% CI 1.1, 4.99), as well as respiratory tract carcinomas (RR = 48, 95% CI 10.72, 214.91). In summary, the majority of RRP were associated with HPV 6 and/or 11. HPV-negative RRP biopsies occurred more frequently in adult-onset patients, and were associated with an increased risk of laryngeal neoplasia and carcinoma in the respiratory tract.

## Introduction

**Figure 1:**
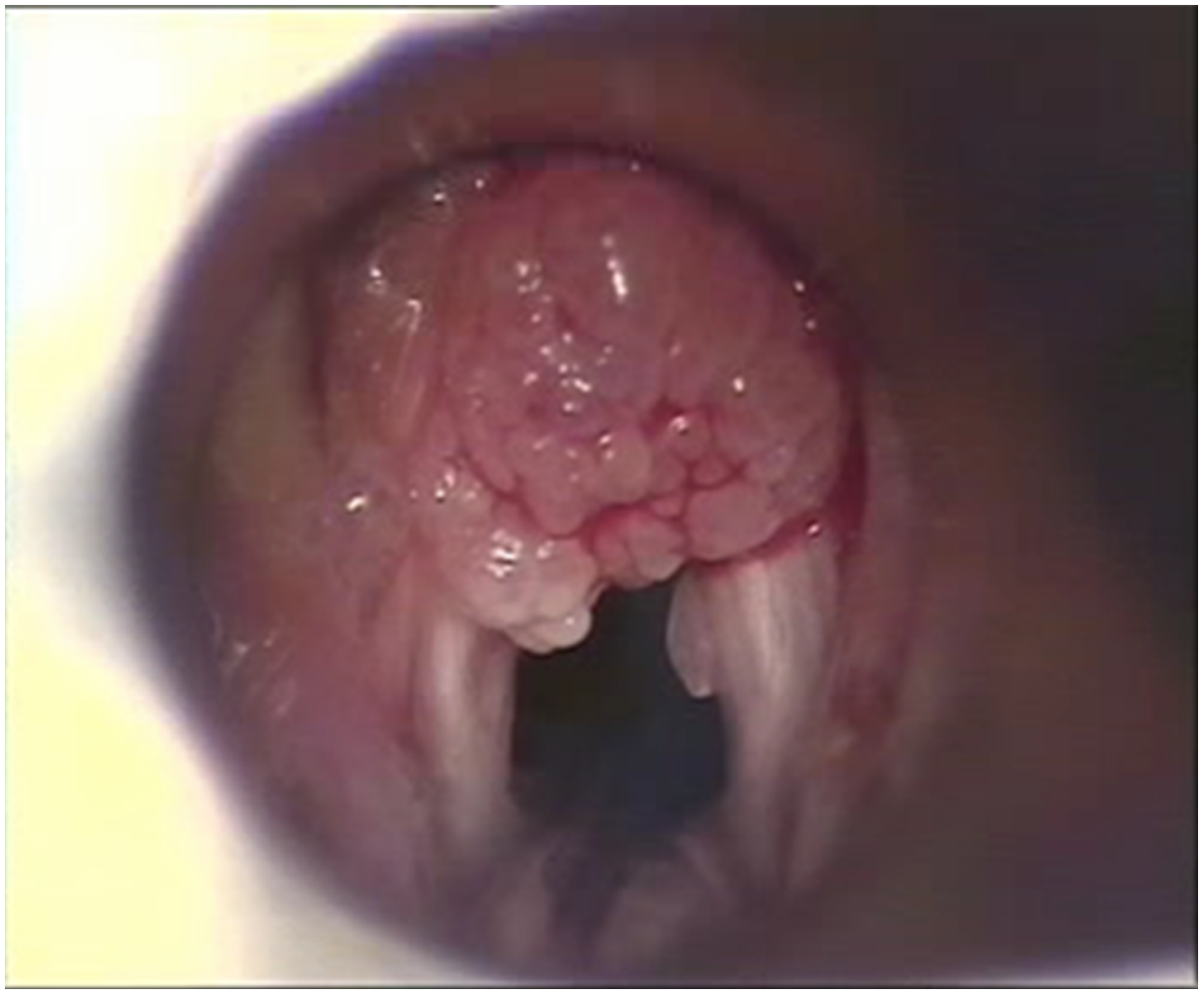
Endoscopic view of laryngeal papilloma in a child with RRP.

Recurrent respiratory papillomatosis (RRP) is a rare condition characterized by recurrent growth of benign papillomas in the respiratory tract (Figure 1), most commonly in the larynx. [1], [2] The disease is categorized as juvenile-onset (JoRRP) if it develops before the age of 18, and adult-onset (AoRRP) for cases that develop after the age of 18. The clinical course is highly variable, with frequent relapses, and may be lifelong. The disease burden is high, often necessitating numerous hospital admissions, but there is no curative treatment. Surgery is required to improve voice quality and to prevent respiratory obstruction. [2], [3] RRP is caused by persistent infection of the respiratory mucosal epithelium by human papillomavirus (HPV), usually HPV 6 or 11. [4]–[8] These ‘low risk’ HPV genotypes (LR-HPV) also cause anogenital condylomas. [9], [10] Condylomas during pregnancy are considered the most important risk factor for acquiring JoRRP by vertical HPV transmission from mother to child. [11] In adults viral transmission may occur during oral sex, but this remains unproven. [12] Re-activation of a latent HPV infection acquired in childhood is another possible cause [13].

LR-HPV may, in rare cases, be associated with malignancy, including carcinoma in the anogenital region. [14] Women with a history of genital warts have an increased risk of cervical intraepithelial neoplasia (CIN) and cervical carcinoma, most likely due to co-infection with ‘high-risk’ HPV genotypes (HR-HPV). [15], [16] Anogenital tract carcinomas and their precursors are linked to infection with HR-HPV, primarily HPV 16 and 18. [17], [18] The proportion of HPV related head and neck carcinomas (HNSCC), particularly oropharyngeal carcinomas, has been rising in recent decades among young adults and in non-smokers. [19]–[23] Similar to all other HPV related cancers, HPV 16 is the most common genotype identified in HNSCC. [24] Although RRP is a benign neoplasm, progression to carcinoma can occur in 3–7% of cases. However, many of these publications are more than 20 years old and based on small series or case reports. [25]–[32] The mechanisms of malignant transformation in RRP, and whether it is preceded by HPV driven precursor lesions, are not clear. [33] Few studies have explored the distribution of HPV genotypes in RRP and the risk of dysplasia and squamous cell carcinoma (SCC) in the respiratory tract. Here, we describe the distribution of HPV genotypes, the prevalence of multiple HPV infections, and the risk of high-grade neoplasia in a cohort of patients with RRP. We also examine whether HPV genotype, gender, age or clinical course are risk factors for developing high-grade neoplasia.

## Materials and Methods

### Ethics Statement

The study was approved by the regional ethical committee of South Eastern Norway Regional Authority. For the use of clinical records, histological review and tissue samples obtained from prior diagnostics of these patients at Oslo University Hospital and Lovisenberg Diaconal Hospital, ethical committee waived the need for informed consent. Letter to each patient, or the next of kin, was however required for the information of the use of specified data and linkage to the Norwegian Cancer registry. [34] Data were anonymized and de-identified prior to analysis and processed according to the requirements of the ethical committee.

### Study Population

Patients from all regions of Norway who were treated at the departments of otorhinolaryngology at Oslo University Hospital and Lovisenberg Diaconal Hospital during 1987–2009 were recruited to the study. [35] Patients were identified in hospital registry systems and their records were reviewed by two laryngologists. Only patients with histopathologically verified laryngeal papillomatosis were included. Clinical records and histological reports from the patients were reviewed. Clinical data obtained from patient records comprised gender, age at disease onset (<18 years, JoRRP; ≥18 years, AoRRP), date of first endolaryngeal procedure and biopsy, number of endolaryngeal procedures, date of last follow-up, and development of dysplasia or SCC in the respiratory tract during the observation period. None of the patients in our cohort had immune deficiencies or were immunocompromised. If patients had several biopsies taken during the observation time, the specimen with highest grade of neoplasia was recorded. The patients’ personal identification number was linked to The Norwegian Cancer Registry [34] to obtain data on high-grade neoplasia in the respiratory tract. Follow-up data were recorded until 1 January 2012.

### Histology Review and Selection of Study Samples

**Figure 2:**
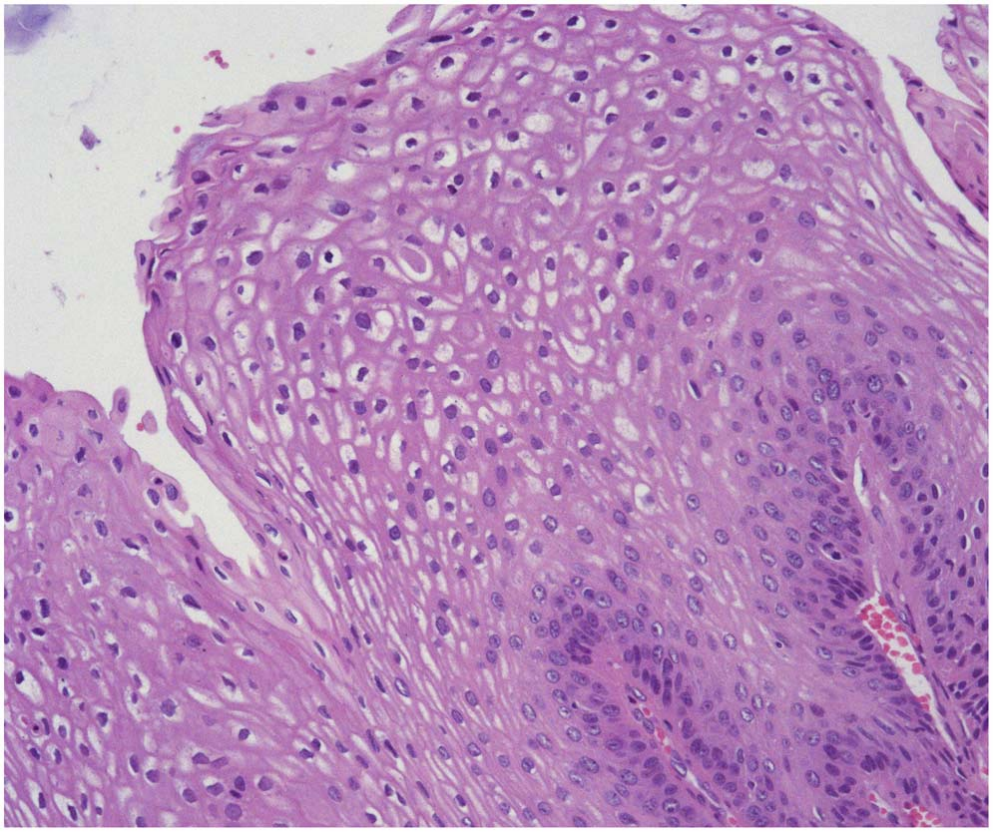
Laryngeal papilloma without atypia in a patient with adult-onset RRP (HEx20).

**Figure 3:**
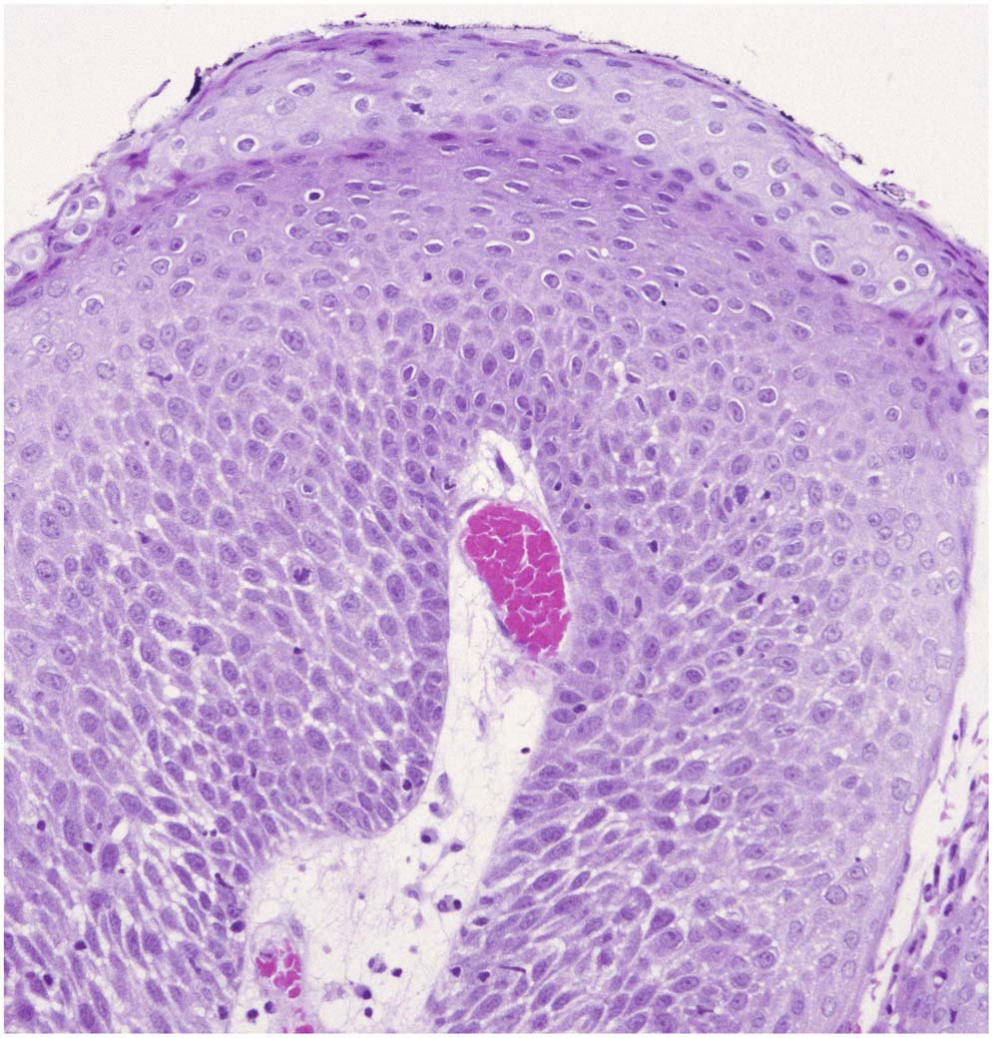
Laryngeal papilloma with moderate dysplasia (SIN 2) in a patient with adult-onset RRP (HEx20).

One biopsy specimen from each patient was selected for blinded histological review according to WHO classification 2005 [36] performed by an experienced head and neck pathologist. One paraffin block with representative tumor tissue was selected for each patient for DNA extraction. The biopsy sample showing the highest grade of neoplasia was selected if available; otherwise, the most recent biopsy specimen obtained during the observation time was selected. Squamous intraepithelial neoplasias (SIN) in the larynx were classified as low-grade (SIN1), moderate (SIN2) or severe (SIN3), equivalent to mild, moderate and severe dysplasia or carcinoma in situ (Figure 2 and 3). For further analyses we categorized the lesions as no atypia/SIN1 (≤SIN1) versus SIN2+, which also included invasive SCC.

### DNA Extraction & HPV Genotyping

DNA was extracted from formalin-fixed paraffin-embedded tissue using proteinase K treatment (56°C, 16 h) with subsequent clean-up using the Biorobot M48 (Qiagen, Hilden, Germany). Quantitative polymerase chain reaction (qPCR) assay was performed on the Roche Lightcycler 480 II using Roche probe master (Roche Diagnostics, Basel, Switzerland) reagents according to the manufacturer’s guidelines. Hydrolysis probes labeled with FAM, HEX and Cy-5 distinguished HPV type 6, 11, 16, 18, 31, 33, 35, 39, 45, 51, 52, 56, 58, 59, 68a and 68b in six multiplex reactions. PCR assays targeting the conserved region E6/E7 in the HPV genome were used. Human genomic DNA was co-amplified and used as internal control. [37] Our method was validated against 92 control samples in the WHO HPV Lab Net Proficiency Panel, and revealed overall agreement in 96.0% of cases. A discrepancy was observed only for HPV 68a, possibly because the primers used were designed for the E6/E7 region, which is not in­cluded for the HPV 68a plasmid in the WHO panel.

### Metagenomic Sequencing

Following whole genome amplification, DNA from HPV-negative samples was sequenced using the Roche GS FLX 454 (Roche Diagnostics, Basel, Switzerland), as described elsewhere [38]–[40], followed by bioinformatic analysis for the presence of viral sequences. In brief, sequence reads were trimmed for tails containing low-quality bases (Phred quality score <20, equivalent to a base calling accuracy of 99% i.e. 1 error in 100 bases). [39], [41], [42] Reads of >80 bp length and <20% ambiguous bases were screened for similarity to human and bacterial DNA, as well as to sequencing reads from negative control samples (water) [41] using an SSAHA2 aligner. [42] Reads with >95% identity to human or bacterial DNA in over 75% of their length were removed. Remaining sequences were assembled to contiguous sequences (contigs) using a WGS CELERA assembler. [43] All assembled contigs and singletons were then compared against GenBank using NCBI BLAST. [44] The raw sequences data are deposited at NCBI GenBank (Bioproject PRJA245067).

### Statistical Analysis

Numerical and categorical data are presented descriptively. Categorical data were analyzed by Pearson’s χ2 test, applying a significance level of 5%. The relative risk with 95% confidential intervals, for developing high-grade dysplasia (including carcinoma) in HPV-negative compared to HPV-positive patients, were calculated using the software package IBM SPSS 18 (USA).

## Results

In total, clinical records and histological reports from 238 patients with RRP were reviewed, from which 14 patients were excluded from the study because paraffin blocks were not available. Hence, the study population comprised 224 patients (174 AoRRP, 50 JoRRP). No significant differences were found in gender, age at diagnosis, duration of observation or development of SIN2+ in the 14 excluded patients compared to the study population. The DNA quality was approved in 221/224 cases (98.7%). Observation time, age at onset and number of surgical procedures are shown in Table 1. The majority of patients were male: 141 (81%) and 31 (62%) of the AoRRPs and JoRRPs, respectively. HPV was detected in 207 (93.7%) of the 221 patients for which DNA quality was approved. All the 207 patients were positive for LR HPV (HPV 6 or 11), comprising HPV 6 in 133/207, HPV 11 in 40/207 cases and HPV 6/11 in 15/207 cases. Co-infection with one or two high-risk HPV types together with HPV 6 or HPV 11 was present in 19/207 patients. In 14/221 patients, HPV was not detectable by qPCR. Metagenomic sequencing of these 14 HPV negative patients found HPV 8 in one case but no virus in the remaining 13 cases. Table 2 shows the difference in HPV genotype distribution between JoRRPs and AoRRPs (p = 0.010) There was a significantly higher rate of patients with co-infection with HR HPV and HPV negative patients in the AoRRPs versus JoRRPs. We also observed a higher rate of HPV 11 infection in children than in adults. Among high-risk HPV types, HPV 33 was the most prevalent type (n = 14) followed by HPV 45 (n = 3), HPV 18 (n = 2), HPV 16 (n = 1), HPV 31 (n = 1) and HPV 35 (n = 1).

**Table 1:** Presentation of the RRP patient cohort (N = 224).

	Juvenile-onset RRP		Adult-onset RRP	
	Median (IQR)	Mean (SD)	Median (IQR)	Mean (SD)
Observation time (years)	12.9 (3.7, 32.9)	20.1 (18.3)	4.0 (0.8, 11.7)	7.8 (9.5)
Age at onset (years)	4.0 (2.0, 6.0)	5.0 (3.8)	34.0 (27.5, 43.0)	36.7 (13.3)
Number of surgical procedures per year*	1.2 (0.5, 3.4)	2.0 (2.1)	1.2 (0.6, 2.6)	2.4 (3.4)
Number of surgical procedures per year during the first three years	2.3 (1.0, 3.8)	2.6 (1.8)	1.0 (0.5, 1.76)	1.2 (1.0)

*During the observation period.

**Table 2:** Distribution of HPV genotype profile in juvenile and adult onset RRP, n (%) (N = 221).

	HPV6*	HPV11	HPV11+6Δ	LR+HR HPV§	HPV negative	Total
Juvenile-onset RRP	25 (51.0)	14 (28.6)	7 (14.3)	2 (4.1)	1 (2.0)	49 (100.0)
Adult-onset RRP	109 (63.3)	26 (15.1)	8 (4.7)	17 (9.9)	12 (6.9)	172 (100.0)

The difference in HPV profile between juveniles and adults was significant (p = 0.010).

*Including one patient with adult-onset RRP who tested HPV 8 positive in metagenomic sequencing.

Δ Infection with both HPV 6 and 11.

§ HPV 6 or 11(LR HPV) in co-infection with one or two high-risk HPV types (HR HPV). High-risk HPV types (HR HPV) comprised HPV 33, 45, 18, 16, 31 or 35.

SIN2+ developed in the larynx in 39 patients (17.6%) during the observation period, with a median time to SIN2+ diagnosis of 2.7 years. The occurrence of SIN2+ did not differ significantly between adult-onset and juvenile-onset RRP patients, nor did time to diagnosis. There was a preponderance of SIN2+ in men, which was significant among the adult-onset patients (p = 0.028) (Table 3). A significantly higher proportion of HPV-negative patients developed SIN2+ in the larynx, compared to HPV-positive patients. Further, the different HPV genotypes did not differ significantly in SIN2+ prevalence (Table 4). Eight (3.6%) patients developed carcinoma in the respiratory tract. Six (2.7%) of these were laryngeal SCC which all had HPV negative papillomas. Two (0.9%) patients had lung carcinomas whose laryngeal papillomas tested positive for HPV 6 and HPV 6/33, respectively. The relative risk of developing SIN2+ in the larynx or carcinoma at any point in the respiratory tract with an HPV-negative biopsy, versus an HPV-positive biopsy, was 2.35 (95% CI 1.1, 4.99) and 48 (95% CI 10.72, 214.91), respectively. Development of laryngeal SIN+ or respiratory tract carcinoma showed no association with LR HPV, HR HPV or clinical course in terms of number of surgical procedures per year for the first three years of the observation period.

**Table 3:** Prevalence of low- and high-grade neoplasia of the larynx during the observation period, n (%) (N = 221).

	Gender	≤SIN 1	SIN 2+Δ	Total N (%)
Juvenile-onset RRP	Male	28 (90.3)	3 (9.7)	31 (100)
	Female	16 (88.9)	2 (11.1)	18 (100)
Adult-onset RRP*	Male	107 (77.0)	32 (23.0)	139 (100)
	Female	31 (93.9)	2 (6.1)	33 (100)

*The difference in development of SIN2+ was significant between genders in adults (p = 0.028).

Δ High grade laryngeal neoplasia, including invasive SCC.

**Table 4:** HPV genotype profile stratified for development of SIN in the larynx, n (%) (N = 221).

Neoplasia	HPV6* n (%)	HPV11 n (%)	HPV6+11 n (%)	HPVLR+HR n (%)	HPVneg n (%)
≤SIN1	114 (85.0)	33 (82.5)	11 (73.3)	17 (89.5)	7 (53.8)
SIN2+ Δ	20 (15,0)	7 (17.5)	4 (26.6)	2 (10.5)	6 (46.2)

The difference in SIN 2+ prevalence between HPV-positive versus HPV -negative patients was significant (p = 0.005).

*Including one patient with adult-onset RRP who tested positive only for HPV 8.

Δ High grade laryngeal neoplasia, including invasive SCC.

In total, 696,218 metagenomic sequences were obtained, but only 0.12% of these were initially classified as viral sequences (803 sequence-reads) with 688 reads showing a viral similarity over their entire length. All of these reads were 99% similar to HPV type 8 and came from a single biopsy sample.

## Discussion

To our knowledge, this is the largest cohort study in RRP patients to examine the HPV genotype profile and determine whether RRP is associated with the development of laryngeal neoplasia and SCC in the respiratory tract. Moreover, the follow-up time was relatively long, and the cohort included both juvenile-onset and adult-onset RRP patients.

All HPV-positive patients were infected by HPV 6 or HPV 11, which is in line with similar studies with the prevalence of HPV 6 or HPV 11 infection to be in the range of 70–98%. [4]–[6] Comparable to RRP, more than 90% of the genital warts contain HPV type 6 (55–90% of cases) or type 11 (5–42%). [45]–[47] In the immunosuppressed individuals, the presence of HPV 11, multiple HPV infections and HR- HPV is higher. [48] In our study the prevalence of HPV-negative papillomas and multiple infections with high-risk HPV was higher in adult-onset than juvenile-onset RRP. None of the adult-onset patients in our cohort had immune deficiencies or were immune decomprised. As far as we know, similar results have not been published.

We observed a higher rate of HPV 11 infection in children than adults. Similar prevalence of HPV 11 in children has also been observed in previous studies. [4], [49], [50] The clinical implication is not clear, but some have postulated a more aggressive clinical course in terms of more extensive growth of the papillomas and hence more frequent need of surgeries. [2], [4], [50], [51] However, we did not find HPV 11 to be associated with laryngeal SIN2+ nor respiratory tract carcinoma, which has been described elsewhere [52], [53].

In our study, 17.6% of patients acquired SIN2+ in the larynx. It is challenging to compare our results with previous studies due to varying terminology and end-points. [54], [55] We applied a dual classification system, which separates low-and high-grade neoplasia, widely used in reporting histopathological findings in the anogenital tract. In a cohort of 129 patients with RRP, Sanchez *et al* [6] reported a total incidence of 13.2% for moderate and severe dysplasias, a figure not dissimilar from our own. Stratified by age groups, we found a significant preponderance of SIN2+ in males within adult-onset RRP. This is probably due to the heavier use of tobacco and alcohol in men than women. Unfortunately, data on such consumption were not available in our study. SCC in the larynx and in the entire respiratory tract occurred in 2.7% and 3.6% of patients, respectively.

This finding is compatible with previous studies in which the prevalence ranged from 3% to 7%. The cumulative risk of laryngeal cancer between 2006 and 2010 in the Norwegian population under the age of 75 was 0.3% in males and 0.1% in females [56], indicating that the risk of laryngeal cancer is higher in patients with RRP than in the general Norwegian population.

We found that a HPV-negative biopsy to be associated with more than a two-fold increase in the risk of developing SIN2+ and almost a 50-fold increase in the risk of SCC in the respiratory tract, when compared to patients with HPV-positive biopsy. A high risk of malignant transformation in HPV-negative patients has also been reported by Lee *et al* [32], who observed a 23.1% prevalence of cancer and an eight-fold increase in the relative risk of malignant transformation in a HPV-negative papilloma versus HPV-positive. In our study, neither HPV genotypes, nor a severe clinical course during the first three years of the observation period, were associated with SIN2+.

Almost 100% of cervical cancers are associated with HPV infection. [57], [58] The association of HPV with HNSCC, mainly pharyngeal carcinomas, is also well documented even though the presence of HPV in these carsinomas varies between 20–80%. [19]–[24], [59] Whether HPV related HNSCC is preceded by HPV driven precursors remains unclear. It is established, however, that HPV negative HNSCC is mainly caused by smoking and alcohol. Among our six HPV-negative patients with laryngeal SCC, there was one daily smoker, two patients who had never smoked, and three patients who had stopped smoking 25 years before SCC was diagnosed. Due to lack of extensive data concerning smoking and drinking habits, further statistical analyses were not possible.

DNA quality was approved in 98.6% of the cases. Since the paraffin blocks were up to 20 years old, a sensitive and robust qPCR method for detecting HPV in paraffin-embedded tissue was used.

This method was validated against a test panel in a HPV reference laboratory. Histology was confirmed in all cases before HPV testing and DNA was only extracted from representative tumor tissue selected by an experienced ear, nose and throat pathologist. Thirteen of the 221 available cases (5.9%) were found to be HPV negative papillomas, which is similar to the rates of HPV negativity reported elsewhere in patients with RRP (4.7%) and anogenital condyloma (5.3%). [6], [47] Other studies have reported detectable HPV DNA in 100% of patients. [4], [60] In general, HPV negativity may represent a genuine finding, but could also represent a false negative due to technical reasons such as sensitivity of the detection method, low viral load, quality of the specimen, loss of the L1 region when HPV is integrated into the human genome, and the presence of unidentified HPV types. Previously unknown HPV types are usually not detected by PCR systems based on known HPV sequences, but the metagenomic sequencing used in our study sequences all the DNA in the sample and uses bioinformatics to analyze whether viruses were present. [38], [39] The detection of HPV is therefore independent of prior knowledge of viral sequences. The number of established HPV types is increasing rapidly: as of March 2014, 184 different HPV types have been identified. [61], [62] In the present study, only one of 14 ‘HPV-negative’ specimens was found to contain virus, suggesting that a small proportion of patients with RRP may indeed be truly HPV-negative.

In each patient the highest grade of dysplasia reported during the time of observation was recorded. Only one biopsy from each patient was revised and used for HPV analysis. 63% of the DNA specimens represented the highest grade of dysplasia found in the patients. Stratifying specimens for whether representing the highest grade of dysplasia or not, revealed similar distribution of HPV profile in these two groups. However, our conclusions regarding association of HPV genotype profile to SIN2+ assume a persistent infection of the same HPV genotype during the clinical course, which is a plausible presumption. [13] Our findings show an increased risk of SCC in the respiratory tract in this RRP cohort, but we are unable to document whether carcinomas are preceded by precursors.

Our finding that HPV negativity occurred more frequently in older male RRP patients and with a higher malignancy potential is novel. It is possible that this association may be confounded by other risk factors, such as smoking and alcohol consumption, for which we had only partial or no data. Notably, there was only one daily smoker among the laryngeal carcinoma patients in this RRP cohort. The potentially carcinogenic effect of HPV exposure was, comprehensively assessed in all patients by qPCR and metagenomic sequencing. The impact of selection and misclassification bias was reduced by histologic review and by linking data to the Norwegian cancer registry.

Analysis of RRP is challenging. It is a rare disease, and hence larger patient cohorts based on multicenter studies are required to confirm our findings.

## Conclusion

In the vast majority of this cohort of Norwegian patients, RRP was associated with HPV 6 and/or 11. While co-infection with HR-HPV types was present in 8.6% of patients, follow-up data revealed no significant association with the development of high-grade SIN or carcinoma. HPV-negativity was rare, occurred more frequently with adult-onset versus juvenile onset RRP and was associated with a high relative risk of laryngeal neoplasia or carcinoma in the respiratory tract. There is an increased risk of cancer in the respiratory tract in RPP patients compared to the general population in Norway.
